# Legislative differences in compulsory mental health care for children and adolescents across European countries

**DOI:** 10.1007/s00787-026-03108-1

**Published:** 2026-06-24

**Authors:** Tjaša Vranetič, Konsta Hakala, Jörg M. Fegert, Maja Drobnič Radobuljac, Riittakerttu Kaltiala, Robert Vermeiren, Astrid Moell, ESCAP coercion and restraint consortium

**Affiliations:** 1 University Medical Centre Ljubljana, Child Psychiatry Unit, University Children’s Hospital, Ljubljana, Slovenia; 2 Faculty of Medicine and Health Technology, Tampere University, Tampere, Finland; 3 Department for Child and Adolescent Psychiatry and Psychotherapy, University Hospital Ulm, Ulm, Germany; 4 University of Ljubljana, Faculty of Medicine, Department of Psychiatry, Ljubljana, Slovenia; 5 University Psychiatric Clinic Ljubljana, Ljubljana, Slovenia; 6 Department of Adolescent Psychiatry, Tampere University Hospital, Tampere, Finland; 7 Vanha Vaasa Hospital, Vaasa, Finland; 8 Child and Adolescent Psychiatry, University of Leiden, LUMC-Curium, Oegstgeest, The Netherlands; 9 Centre for Psychiatry Research, Department of Clinical Neuroscience, Karolinska Institutet, & Stockholm Health Care Services, Region Stockholm, Stockholm, Sweden

**Keywords:** Compulsory mental health care, Child and adolescent psychiatry, Mental health legislation, Involuntary hospitalisation, Coercion

## Abstract

There is growing concern about the protection of children’s rights in compulsory psychiatric care. To examine procedural and legal variations in compulsory mental health care for children and adolescents across European nations, the European Society for Child and Adolescent Psychiatry (ESCAP) commissioned this study. A structured questionnaire was developed using Google Forms and distributed via email to representatives of all 36 ESCAP member countries. The survey gathered information on legislation, commitment criteria, and procedural requirements for compulsory mental health care in minors. Data were analysed qualitatively to identify common themes and notable legislative differences. A total of 35 responses were received, covering 24 countries. 29% of participating countries reported child-specific legislation for involuntary hospitalisation of minors, with key differences from adult legislation concerning commitment criteria, involvement of legal guardians, and considerations regarding minors’ capacity to consent to treatment. A mental disorder diagnosis is required for involuntary admission in 88% of countries, with 52% of those countries specifying severe mental disorder. Further, 71% of countries require that the person pose a danger to themselves or others. The legal age of consent for treatment varies widely (12–18 years), with 92% of countries allowing appeals against involuntary hospitalisation by the minor or their guardians. The observed legislative disparities underscore the varied approaches to compulsory mental health care for minors in Europe, suggesting a need for developing shared guidelines to promote equitable and effective care across countries.

**Supplementary Information:**

The online version contains supplementary material available at https://doi.org/10.1007/s00787-026-03108-1.

## Introduction

Compulsory mental health care entails psychiatric treatment without the patient’s explicit consent, typically when a mental disorder severely impairs the person’s decision-making capacity. It includes *involuntary hospitalisation*, where individuals are detained in psychiatric facilities, and *outpatient commitment*, where treatment is mandated in the community. While such measures at times may be necessary and life-saving, they raise important human rights concerns [1, 2] and stand in contrast to standard mental health care which emphasises collaboration, autonomy, and shared decision-making [3]. When applied to children and adolescents, compulsory care requires particular scrutiny, as emphasised by the UN Convention on the Rights of the Child [4], which calls for strong justification and safeguards when involuntary treatment involves minors. Additionally, minors’ vulnerability arises not only from developmental differences and evolving decision-making capacity [5], but also from the fact that parents or legal guardians may consent to treatment on their behalf [6].

The term ‘minors’ is used in this paper interchangeably with ‘children and adolescents’ and refers exclusively to individuals under the age of 18 years.

In the adult context, involuntary psychiatric treatment is governed by diverse legal frameworks [7, 8], each jurisdiction outlining specific conditions (‘the commitment criteria’) for admission without consent. While the presence of a mental disorder is typically required (in some jurisdictions, that disorder must be classified as severe or as mental illness), additional criteria often include the need for treatment (when the individual’s condition is likely to deteriorate without intervention) and/or the potential dangerousness of the person to self or others [9]. A comprehensive survey conducted by the Ethics Committee of the European Psychiatric Association across 40 member countries [10] revealed substantial variation in legal procedures, including the involvement of non-medical actors, such as judges and legal representatives, in admission decisions. While dangerousness remains a common justification for compulsory psychiatric care, the authors emphasised the value of explicitly including medical necessity (the need for treatment criterion) in legislation to promote therapeutic rather than purely protective approaches, to help reduce stigma and promote patient-centred care.

In the context of child and adolescent psychiatry, a scoping review by Schölin et al. [6] examined mental health detention practices and legislative differences in 14 Western countries, suggesting approximately 20% of psychiatric admissions among minors were involuntary, with considerable variation in the criteria used across jurisdictions. The study identified a critical knowledge gap: a lack of research on different forms of detention and their application within human rights frameworks. The authors called for more detailed investigations to better understand and address disparities in the involuntary treatment of minors.

To work towards a more ethically grounded and equitable health care system, the European Society for Child and Adolescent Psychiatry (ESCAP) commissioned a study on legislation in Europe regarding compulsory mental health care for children and adolescents.^1^ Following Wasserman et al. [10], the aim of this study was to map and synthesise legislative and procedural frameworks governing compulsory mental health care for minors in European countries.

## Methods

A Google Forms questionnaire was created through collaborative meetings with the ESCAP Policy Division and ESCAP Executive Board members from autumn 2023 to spring 2024. A draft version was pilot tested in February 2024 with four professionals in the field. Based on their feedback, minor adjustments were made to improve clarity. The final version of the questionnaire (Online Resource 1) included structured and open-ended questions addressing legislation and clinical practices related to involuntary hospitalisation and outpatient commitment.

The questionnaire was distributed in March 2024 via email to representatives of all 36 ESCAP member countries, using the ESCAP contact person mailing list. Representatives—all affiliated with national child and adolescent psychiatry organisations—were asked to delegate one or more professionals with the most experience in child and adolescent psychiatry or mental health law to complete the questionnaire. Reminder emails were sent one month and three months after the initial invitation. Altogether, the survey received 35 responses (from March 2024 to November 2024), covering 24 European countries (some countries had two or more respondents). Follow-up contact was made with selected respondents to clarify specific aspects of their country’s legislation where needed.

Based on the collected data, two manuscripts were developed: the present one, which focuses on legislative differences concerning involuntary hospitalisation, and a second manuscript about legislative variations in the use of coercive measures [11].

Descriptive statistics were used to summarise structured responses. Open-ended responses were analysed using a deductive qualitative content analysis approach to identify variations and patterns in legal frameworks and practices. Coding and thematic organisation were conducted manually by the research team. Draft versions of both manuscripts were submitted to the respondents for feedback.

Because the study is based on expert survey responses rather than independent statutory analysis, country-level descriptions reflect respondents’ interpretations of applicable law and practice. While follow-up clarifications and respondent feedback on manuscript drafts were obtained, the legal characterisations presented should be understood as expert-informed summaries rather than formally verified legal determinations.

## Results

Respondents represented 24 different countries: Austria, Belgium, Bulgaria, the Czech Republic, Denmark, Estonia, Finland (2 respondents), France, Germany (2 respondents), Greece, Hungary, Israel, Italy (5 respondents), Luxembourg, the Netherlands (4 respondents), Norway, Portugal, Republic of Serbia, Slovenia (2 respondents), Spain (2 respondents), Sweden, Switzerland, Ukraine, and Great Britain^2^. Most respondents were child and adolescent psychiatrists with a median of 21 years of experience in the field (Table 1). Following the initial drafting of the manuscripts, an additional expert from Switzerland was included during the revision process.

A summary of all responses for each country is available in Online Resource 2.

**Table 1:** Characteristics of respondents (*n* = 35) to a survey on compulsory mental health care legislation for children and adolescents in ESCAP member countries

ESCAP member countries represented, *n* (%)	24 (67%)
Years of experience	2–35 years (median 21 years)
*Profession*	
Child and adolescent psychiatrist, n (%)	32 (91%)
Psychologist, n (%)	2 (6%)
Psychiatrist, n (%)	1 (3%)
*Academic/leading position*	
Professor, n (%)	5 (14%)
Head of department / Medical director, n (%)	11 (31%)

ESCAP = European Society for Child and Adolescent Psychiatry

### Separate legislation regarding involuntary hospitalisation for minors

Less than a third (29%) of the participating countries have a separate legislative framework governing the involuntary hospitalisation of minors, distinct from that applied to adults (Table 2). The reported differences concerned four main areas: (1) commitment criteria, (2) procedural and treatment safeguards, (3) regional legal frameworks, and (4) the legal authority responsible for ordering compulsory care. A summary of the general commitment criteria across all countries, including those without age-specific legislation, is presented in a separate section below.

**Table 2:** Existence of separate legislation for involuntary psychiatric hospitalisation of minors, according to professionals from 24 ESCAP member countries

	*n* (%)	Countries
Yes	7 (29%)	Belgium, the Czech Republic, Finland, Germany, Hungary, Israel, Luxembourg
No	17 (71%)	Austria, Bulgaria, Denmark, Estonia, France, Great Britain, Greece, Italy, the Netherlands, Norway^b^, Portugal, Republic of Serbia, Slovenia, Spain^a^, Sweden, Switzerland^b^, Ukraine

ESCAP = European Society for Child and Adolescent Psychiatry

^a^ In Catalonia, all psychiatric admissions of minors require judicial authorisation; parental consent is insufficient, and clinicians must notify a judge within 24 h, making voluntary admission legally impossible

^b^ In Switzerland and Norway, no separate legislation for minors exists, however, its application varies depending on age, as described in the text

*Commitment criteria* were reported to be either stricter or more lenient for minors than for adults, depending on the country. In Belgium, a stricter requirement applies to minors in that a medical indication (diagnosis of a mental disorder) is always required for involuntary admission, whereas this is not explicitly required for adults. In Israel and Finland, the diagnostic threshold is lower for minors: any mental disorder (Israel) or a severe mental disorder (Finland), compared to psychotic disorders for adults. However, additional criteria for commitment are stricter for minors in Finland, requiring other treatment options to be inadequate.

In contrast, minors in Luxembourg and Switzerland may be admitted involuntarily under family law without a psychiatric diagnosis, based on concerns for the child’s welfare. Similarly, in the Czech Republic, minors may be hospitalised involuntarily in emergencies involving suspected serious abuse or neglect of the minor. In all three countries, a psychiatric diagnosis is required for involuntary admission in adults.

Norway has a distinct legislative approach in which minors under the age of 16 cannot be involuntarily admitted under mental health legislation. Admission in these cases requires guardian consent and is formally classified as voluntary. For children aged 12 years and above who do not agree to admission, the case must be reviewed by an independent control commission. From the age of 16, minors may be hospitalised involuntarily under the same provisions as adults.

More extensive *procedural or treatment safeguards* are applied for minors in some countries. In Hungary, involuntary hospitalisations of minors are subject to stricter court oversight. Cases must be reviewed regularly to assess whether the level of risk to the minor or others continues to justify admission. In Slovenia—although legislation does not formally distinguish between adults and minors in terms of involuntary admission—specific restrictions apply to minors once hospitalised. These include a prohibition on admission to adult wards and restrictions on certain treatment modalities, such as electroconvulsive therapy, hormone therapy for mental disorders, and medication doses exceeding approved limits. Similarly, in Austria and Sweden, although the same legislation governs both adults and minors, it includes a section outlining specific provisions applicable to minors.

Several countries reported *regional legal frameworks* that differed between minors and adults. In Belgium, Germany, Spain and Switzerland, national legislation on involuntary hospitalisation applies to both adults and minors, but is complemented by regional or cantonal laws that introduce specific provisions for minors. These may include differences in procedural oversight, required documentation, or permissible interventions.

Differences were also observed in the *legal authority responsible for ordering involuntary hospitalisation*. In Germany, most minors who need acute psychiatric care but are unable to consent are admitted involuntarily through parental applications to the family court, rather than under the general mental health act. Similarly, in Luxembourg, minors are admitted through juvenile courts under family law, while adults fall under the jurisdiction of civil courts. In Belgium, minors cannot be admitted under criminal law, unlike adults.

### Minor’s right to decide upon treatment and the role of parents/legal guardians

The age at which minors can consent to medical and psychiatric treatment varies across countries, ranging from 12 to 18 years where a fixed age is defined (Table 3). In 33% of the countries, no specific legal age of consent is set; instead, each case is evaluated individually based on the child’s developmental level.

**Table 3:** Age of consent for medical and psychiatric treatment of minors, according to professionals from 24 ESCAP member countries

	Countries
*Fixed minimum age of consent*	
14	Austria, Hungary, Ukraine
15	Denmark, Israel, Republic of Serbia, Slovenia
16	Great Britain^b^, Norway, Spain^a^
18	Bulgaria, France, Germany, Greece, Italy
*Tiered system*	
- Under 12: parents decide - 12–15: joint decision - 16 and over: minors decide	the Netherlands
*No fixed age of consent*	
Individual assessment	Belgium, the Czech Republic, Estonia, Finland, Luxembourg, Portugal, Sweden, Switzerland

ESCAP = European Society for Child and Adolescent Psychiatry

^a^ In Spain, the general minimum age for consent is 16, but parental involvement is required until 18 in high-risk cases. Minors under 16 may consent if assessed as sufficiently mature

^b^ In Great Britain, minors under 16 may consent to treatment independently if deemed ‘Gillick’ or ‘Fraser’ competent, meaning they understand, retain, and evaluate the information and can communicate an informed decision

The legal role of parents (or legal guardians) in the involuntary psychiatric treatment of minors also varies considerably across countries. In many countries, parents may give consent for admission when the child is under the legal age of consent, or until the child is deemed competent to decide. Some countries grant parents full decision-making authority until the minor turns 18, while others introduce increasing autonomy from age 12 onwards, with joint decision-making required in some cases.

In most countries, parents or legal representatives can request or initiate hospitalisation, but medical or judicial review is required for involuntary measures. In some countries, parental consent may be legally sufficient to authorise involuntary hospitalisation, although these procedures are in practice discouraged (Portugal, Republic of Serbia, Great Britain). A few countries allow involuntary hospitalisation of minors against parental wishes if deemed medically necessary (Denmark, Estonia, Finland, Sweden), if decided by judicial authority (Belgium, Luxembourg, Switzerland) or social services/family courts (Slovenia). In special cases, some countries permit hospitalisation based solely on parental consent, with admission classified as voluntary regardless of the child’s consent—for example, in cases involving very young children (approximately under the age of 10 in Sweden and under 12 in the Netherlands and Finland) and in cases of patients with chronic lack of capacity to consent (Switzerland). In Luxembourg, when a minor is involuntarily admitted, parents automatically lose parental authority, which is then granted to the hospital’s general director.

### Commitment criteria for involuntary psychiatric hospitalisation

The commitment criteria for involuntary psychiatric hospitalisation in the participating countries typically consist of a combination of a basic requirement—the presence of a mental disorder (in 88% of countries)—and additional conditions that vary across jurisdictions. A detailed description of commitment criteria by country is available in Online Resource 2.

In 46% of countries, the mental disorder must be classified as severe in order to justify involuntary admission. In contrast, Luxembourg, the Czech Republic and Switzerland permit involuntary hospitalisation in the absence of a psychiatric diagnosis—for example, in cases involving child protection or public safety (as described in the section above).

Dangerousness to self or others is a second requirement in a majority of countries (71%), with a clinically perceived causal link between the mental disorder and the assessed risk usually required. In Israel, Luxembourg and the Netherlands, the risk of harm can also be towards normal development.

Only 29% of the countries include the need for treatment as a legal requirement for involuntary hospitalisation. In 43% of these countries, the criterion is applied disjunctively, meaning it can be used as an alternative to the dangerousness requirement.

Other additional requirements include impaired judgement due to a mental disorder, resulting in an inability to provide informed consent (33% of countries), and situations where all other treatment options have been determined to be unsuitable or insufficient to avert harm or meet the child’s need (42% of countries). 

### Administrative process of involuntary hospitalisation

All respondents described some external party (e.g. independent physician, judge, lawyer, or administrative court) involved in the assessment and decision-making process regarding involuntary hospitalisation of a minor. The main variation lay in when these external parties become involved. In most countries (79%), a judge or court is involved early in the process, either deciding on the admission itself or reviewing its justification within the first few days. In other countries, judicial review occurs only after the initial period of hospitalisation (Online Resource 2).

The duration of involuntary hospitalisation is determined by an administrative court in most countries, although in a minority of jurisdictions it is based on the clinical judgement of a physician. While most countries do impose a maximum duration for the initial admission, it ranges from 48 h to 1 year, typically with the possibility to extend following judicial and/or medical reassessment. Luxembourg is the only country where the duration of admission is indeterminate and not subject to mandatory reassessment. 

### Possibility to appeal involuntary hospitalisation

In 92% of participating countries, involuntary hospitalisation can be appealed and overturned. Only Bulgaria and Ukraine reported no available appeal mechanism; however, in Ukraine, patients may pursue legal action against the hospital after discharge.

The right to appeal is most commonly granted to the patient (Table 4). In many countries, parents or legal guardians may also appeal, either jointly with the minor or on their behalf. In a few jurisdictions, other actors such as legal representatives, patient advocates, or psychiatric institutions may also file an appeal. In Norway, the control commission (appointed by the Ministry of Health) monitors patient welfare and may initiate or support appeals based on concerns raised by patients, relatives, or staff. In Austria, a consultant psychiatrist may submit a counter-appeal in response to an appeal filed by the patient.

**Table 4:** Right to appeal involuntary psychiatric hospitalisation of minors, according to professionals from 24 ESCAP member countries

	Country	Who has the right to appeal
Yes, *n* = 22 (92%)	France, Sweden	Patient
	Austria, Denmark, Finland, Israel, Republic of Serbia, Spain	Patient, parents/guardians
	Belgium, Italy, Norway, Switzerland	Patient, parents/guardians, other relatives
	Estonia	Patient, legal guardian, close family member, other interested party, state-appointed defender
	Luxembourg	Parents/guardians, legal representative
	Great Britain	Patient through legal representative, nearest relative
	Germany, Hungary, Slovenia	Patient through legal representative (Germany: guardian ad litem; Hungary: parent/guardian/lawyer; Slovenia lawyer)
	the Netherlands	Patient, legal representative, care provider
	Portugal	Patient, person of trust, the appointed defender, whoever has legitimacy to request involuntary hospitalisation
	the Czech Republic, Greece	Not answered
No, *n* = 2 (8%)	Bulgaria, Ukraine	-

In some countries, a legal representative is appointed to act in the minor’s best interests and may initiate an appeal, such as a *guardian ad litem* in Germany. Several countries also offer designated legal counsel to support the patient through the appeal process. Denmark, Estonia, Great Britain, the Netherlands, Slovenia, and Sweden provide such support, with the Netherlands offering legal counsel as soon as the decision on involuntary hospitalisation is made. In contrast, 71% of countries do not report any formal support mechanisms for minors seeking to appeal.

In most participating countries, appeals against involuntary hospitalisation are handled by an administrative court, however, the appeal mechanisms vary across countries. In some, the appeal is handled within the psychiatric care system itself—for example, by the medical director (the Netherlands) or by an independent committee (Norway, Israel). In Israel, both the court and an independent committee may be involved. In Slovenia, appeals against involuntary hospitalisation are directed to the court, while decisions on specific measures or treatment may be appealed directly to clinical staff (e.g., physician, nurse, ward director) or indirectly through patient rights representatives. In Germany, all patients have a personal hearing with a judge as part of the appeals process. In Luxembourg, the appeal is reviewed by the same judge who issued the initial order for involuntary treatment.

### Facilities for treatment

Almost all countries (83%), apart from Belgium, Estonia, Hungary and Ukraine, reported treating children and adolescents who were involuntarily hospitalised in psychiatric units with doors locked at all times, with some reporting only having these units (the Czech Republic, Luxembourg). However, most of the participating countries have a combination of secure units, occasionally locked units, and open units (unlocked doors at all times), with some also using paediatric units as well as adult psychiatric units. In Belgium, children and adolescents can also be involuntarily hospitalised to a secure confinement service for ‘legally irresponsible adults’ for the treatment of psychiatric disorders. In Wales (Great Britain), there are no secure wards for children and adolescents, if needed, children are transferred to England.

### Outpatient commitment

Most participating countries (79%) allow outpatient commitment for the treatment of mental disorders in children and adolescents. Only a minority of countries (Estonia, Finland, Germany, Spain and Ukraine) do not permit this form of compulsory care.

## Discussion

This study provides an overview of the legislative frameworks regarding involuntary psychiatric hospitalisation of children and adolescents in 24 European countries. Drawing on expert insights from 35 professionals, predominantly experienced child and adolescent psychiatrists, the study highlights the diversity of compulsory mental health care legislation for minors across Europe. While some countries have separate legislation for children and adolescents, a majority apply general adult mental health laws. Commitment criteria, age of consent, procedural safeguards, involvement of external authorities, appeal rights and availability of specialised facilities vary considerably. While most countries require a mental disorder diagnosis and perceived dangerousness of the patient, only a minority include the need for treatment as a criterion, reflecting different national approaches to involuntary treatment in child and adolescent psychiatry.

The widespread use of adult mental health legislation for minors raises concerns about whether children’s developmental needs and evolving capacities are adequately recognised [4, 12]. While some countries incorporate minor-specific safeguards within broader legal frameworks, few provide the comprehensive protection that specific legislation might offer. Adult-oriented laws often lack mechanisms to assess and respect a minor’s evolving capacity [13], which may inadequately balance parental involvement with the child’s autonomy [12, 14] and fail to ensure that minors receive essential procedural safeguards, including access to legal representation when subject to involuntary treatment.

Importantly, child-specific legislation does not inherently guarantee stronger rights protection. Our findings indicate they can either enhance or restrict minors’ rights: stricter criteria may raise the threshold for involuntary admission of minors, while expanded protective authority may facilitate detention without a formal psychiatric diagnosis or on broader welfare grounds. These contrasting approaches highlight that child-specific legislation must be assessed by how it balances care, protection, and autonomy. Fundamentally, these variations reflect a lack of consensus within Europe on whether a child’s best interest is best served by protection or autonomy.

Our findings reveal substantial variations in how European countries interpret and regulate children’s decision-making capacity. International legal and medical frameworks increasingly emphasise children’s autonomy alongside access to care [4, 15, 16], and research suggests decision-making competence may emerge around age 12 [5], supporting a shift from fixed age thresholds to individualised assessments [17, 18]. However, our findings suggest legal inconsistencies concerning the age of consent, raising questions about formal equality (i.e. the identical application of rules to all) and equity (i.e. proportionate decision-making responsive to children’s evolving capacities). A strict age threshold may promote formal equality by applying the same rule to all individuals at a given age but overlook differences in developmental maturity, cognitive capacity, and individual circumstances—thus falling short of equity, which requires tailoring approaches to diverse needs [19]. Conversely, frameworks without fixed age limits may better align with equity by accommodating individual differences, but risk undermining adolescents’ autonomy if assessments of maturity are inconsistently applied or overly cautious. This may result in young people never fully attaining the right to consent, especially in coercive contexts.

Treating all minors as young children risks undermining their agency, while conversely treating them as if they possess full adult capacities may neglect the special protections mandated by the UN Convention on the Rights of the Child [4]. Both approaches fail to recognise the distinct legal and developmental status of minors. Striking a balance between ensuring equitable treatment and safeguarding a minimum standard of rights for all youths remains a key ethical and legal challenge in the regulation of compulsory psychiatric care.

Similarly to variations in the legal age of consent, parental involvement in psychiatric admissions differs substantially across countries. These differences mean that a child of the same age may be treated as fully autonomous in one country but entirely dependent on parental consent in another. Moreover, in some countries, hospitalisation approved solely by parents is still considered voluntary in some cases, even if the child objects, blurring the line between consent and coercion and raising the risk of informal deprivation of liberty. This creates a conceptual grey zone in the classification of admissions: while parental authority may be appropriate for young children or those lacking decision-making capacity, classifying parent-authorised admission as ‘voluntary’ when an adolescent with decision-making capacity objects becomes ethically problematic. Such admissions bypass formal involuntary procedures with associated judicial safeguards, yet involve deprivation of liberty against the individual’s will. This ambiguity may deprive adolescents of procedural protections otherwise guaranteed under formal involuntary admission frameworks.

The commitment criteria vary substantially across countries. While most jurisdictions require a diagnosis of a mental disorder, the Czech Republic, Luxembourg and Switzerland permit involuntary admission of a minor without such a diagnosis. Given that involuntary hospitalisation is among the most intrusive interventions—entailing loss of liberty and often experienced as frightening and distressing [20]—its use without a clear diagnostic foundation raises serious ethical and legal questions. Such practices risk bypassing safeguards established by international frameworks such as the UN Convention on the Rights of the Child [4] and the UN Convention on the Rights of Persons with Disabilities [2], potentially leading to violations of children’s fundamental rights to liberty and protection from arbitrary detention.

Consistent with studies on adult legislation [10, 21], we found most jurisdictions use a *dangerousness* criterion, with far fewer requiring a criterion of *need for treatment.* Stressing dangerousness as a requirement for intervention may reflect an emphasis on public protection [22], combined with concerns about self-harm or suicide, but risks reducing psychiatric care to crisis management, thereby limiting opportunities for early intervention. It may also reinforce public misconceptions that associate mental illness with violence, contributing to social stigma [23]. By contrast, incorporating the need for treatment criterion could allow for a more non-stigmatising and patient-centred approach [10], enabling psychiatrists to address mental health concerns before they escalate to crisis levels.

Procedural and administrative processes surrounding involuntary hospitalisation vary considerably across countries, raising concerns about unequal protections and inconsistent respect for the rights of children. Involvement of non-medical actors (such as legal or administrative authorities) may safeguard children’s rights but can also introduce delays or decisions influenced by factors beyond clinical judgment [10]. Maximum admission duration (before mandatory reassessment or renewal) ranges from a few days to one year. While many countries impose such limits to ensure periodic review, Luxembourg lacks compulsory reassessment procedures, thereby increasing the risk of prolonged or insufficiently scrutinised detention. Uneven access to appeals or independent legal representation raises concerns about procedural fairness, especially for minors who may lack the resources or capacity to advocate for themselves [24]. Additionally, differences in treatment settings, including use of adult wards, which may be inappropriate for minors [25], highlight disparities in rights protection and adherence to the international human rights standards and the principle of the best interests of the child.

### Strengths and limitations

Our study includes responses from 24 European countries, offering a broad comparative overview of involuntary psychiatric treatment of minors. The involvement of experienced professionals and a pilot-tested questionnaire enhanced clarity and relevance, while follow-up clarifications improved accuracy and transparency.

However, several limitations must be acknowledged. As the data are based on expert opinion rather than direct legal analysis or observational data, the potential for misinterpretation or bias exists, particularly where national legislation is complex or where laws vary within the country. Inaccuracies in describing complex legal frameworks may occur when relying on respondent interpretation. This risk is particularly relevant for countries with decentralised systems or parallel legal pathways (e.g., mental health law vs. family law). While we have taken steps to verify ambiguous responses and have revised country descriptions where needed, our findings represent respondents’ understanding and interpretation of applicable law and practice, rather than independent statutory legal analysis. This interpretative frame should be considered when drawing conclusions from the comparative data.

Additionally, our study focused on legislation governing involuntary treatment within healthcare settings, e.g. psychiatric hospitals. However, some countries have separate provisions that allow minors to be involuntarily placed in other institutions or closed residential facilities, for example due to behavioural disorders or intellectual disabilities. These parallel systems, not examined here, may operate under different legal standards, adding to the complexity of involuntary care for minors.

Moreover, some countries were not represented, limiting the generalisability of findings across all ESCAP member states. Their absence may reflect limited resources or less-developed legislative frameworks regarding child and adolescent mental health care, underscoring the need for equitable policy development and cross-national collaboration. While not fully comprehensive, this study offers a valuable overview of current legal practices and identifies key areas for improvement.

### Policy and practice implications

Our findings underscore notable variations in the legal frameworks governing involuntary psychiatric treatment of minors across Europe, highlighting differences in legal safeguards and the extent to which developmental differences are considered. These differences should be interpreted in context, recognising that some variation may reflect differing legal traditions, cultural values and health system structures across countries. A single uniform legislative model for involuntary treatment of minors across all jurisdictions may therefore neither be feasible nor desirable. Instead, our findings support the identification and strengthening of core minimum safeguards, such as protections against arbitrary deprivation of liberty, ensuring mandatory time-limited review of admissions, and providing accessible mechanisms to appeal involuntary admission, aligning with the UN Convention on the Rights of the Child. Establishing such baseline protections could strengthen the protection of children’s rights and autonomy across jurisdictions while still allowing flexibility in how national systems are structured and implemented. At the same time, the question of where universal minimum safeguards should be drawn—and by whom—remains an open issue that extends beyond the scope of this study. Importantly, whilst judicial oversight may strengthen protections, legal mechanisms should complement rather than substitute the ethical and clinical responsibilities of clinical teams.

## Conclusions

This study provides a comparative overview of how European countries regulate involuntary psychiatric care for minors, revealing substantial cross-national variation in legal frameworks and safeguards. Our findings identify structural disparities in key procedural and rights-based protections that may undermine the consistent protection of children’s rights and point to a shared ethical responsibility: to ensure that all children and adolescents, regardless of jurisdiction, are afforded legal protections that reflect their developmental needs and uphold their autonomy. Future efforts should focus on developing shared clinical and ethical guidelines that respect national legal contexts while aligning more closely with international human rights standards.

## Supplementary Information

Below is the link to the electronic supplementary material.

Supplementary Material 1

Supplementary Material 2

## Data Availability

The data supporting the findings of this study are available from the corresponding author upon reasonable request.

## Footnotes

1 In 2020–2021, the ESCAP Policy Division, then chaired by current President Jörg Fegert, conducted a survey among member societies to identify key policy priorities. One of the main concerns raised was the use of restraint and coercive measures. In response, under the leadership of Maja Drobnič Radobuljac, the Policy Division has concentrated on this issue and is hereby now publishing the results to address members’ concerns.

2 The response from Great Britain includes England and Wales but not Scotland.
