# Supplementary material for: Legislative differences in compulsory mental health care for children and adolescents across European countries
Source: Eur Child Adolesc Psychiatry. 2026 Jun 24;35(11):3439–48. doi: 10.1007/s00787-026-03108-1 (PMC13710461; doi:10.1007/s00787-026-03108-1)
Supplement: Supplementary file 1 [file 787_2026_3108_MOESM1_ESM.pdf]

Online resource 1

Supplement to ’Legislative Differences in Compulsory Mental Health Care for Children and Adolescents Across European Countries’ by Vranetič et al. 2025

Survey on compulsory psychiatric treatment for children and adolescents in EU

Dear colleague,

As part of the ESCAP initiative, we want to identify the current legislation and practice regarding the compulsory psychiatric treatment of children and adolescents in the countries of the European region. The survey will serve as a basis for recommendations to support the rights of children undergoing treatment for mental illness.

You have been selected by your peers as one of the experts in this field. Your expertise and insights are invaluable to us. We cordially invite you to participate in this survey. The duration of the survey depends on the complexity of the laws and practices in your country. The survey is not anonymous, as we may need to contact you if we require further information. Therefore, we also offer you the opportunity to join the expert consortium for possible future publications of the data obtained through this survey.

SECTION 1

Name:

E-mail address (We may contact you for further clarifications, if needed):

Country (and state if there is different legislation for different states within country):

Profession:

Years of experience in child and adolescent psychiatry:

In which position (relevant to the topic of the survey) do you currently work?

Workplace:

- o Outpatient
- o Hospital

Do you have experience with involuntary psychiatric treatment of adults?

- o Yes
- o No

Do you have experience with involuntary psychiatric treatment of children and adolescents?

- o Yes
- o No

Would you like to be a part of the expert consortium for possible future publications of the data obtained through this survey? If so, please give us your relevant affiliations as you would like them to be stated.

SECTION 2

Please answer the following questions regarding the situation in your country. If there are some aspects you feel are not captured with our questions, please use the free text question at the end of the questionnaire to elaborate. If possible, add links to the online laws you are referring to.

1. Does legislation in your country define at what age children and adolescents have a right to decide upon medical/psychiatric treatment? Or are there any other criteria to decide the capacity of children and adolescents to give consent? Please describe and possibly add links to the legislation.

2. Is there legislation regulating treatment against the patient's will for psychiatric disorders in children and adolescents in your country? If yes, please write the name of law/laws with English translation and possibly add links to the laws online.

3. What facilities are available for involuntary hospitalization for the treatment of psychiatric disorders in children and adolescents in your country?

- Secure/intensive/locked psychiatric ward for children/adolescents (locked doors at all times)
- Secure/intensive/locked psychiatric ward for adults (locked doors at all times)
- Optionally secure/intensive/locked wards for children/adolescents (doors can be locked when needed)
- Optionally secure/intensive/locked wards for adults (doors can be locked when needed)
- Open child and adolescent psychiatric wards (the doors are not locked)
- Pediatric wards
- Other:

4. What facilities are available for the use of seclusion (keeping the person away from others; e.g. in an isolated room) and restraint (restraining the patient's movements by holding or fixating their waist and/or hands and ancles to a bed by belts/cuffs, or alike mechanical measure) for the treatment of psychiatric disorders in children and adolescents in your country?

- Secure/intensive/locked psychiatric ward for children/adolescents (locked doors at all times)
- Secure/intensive/locked psychiatric ward for adults (locked doors at all times)
- Optionally secure/intensive/locked wards for children/adolescents (doors can be locked when needed)
- Optionally secure/intensive/locked wards for adults (doors can be locked when needed)
- Open child and adolescent psychiatric wards (the doors are not locked)
- Pediatric wards
- Other:

SECTION 3: Coercive measures

Measures applied "against the patient's will or in spite of his or her opposition"

5. Which coercive measures can be used in psychiatric inpatient care for children and adolescents in your country?

- Forced medication (e.g. giving an injection against the patient's will)
- Forced feeding (i.e. nasogastric tube-feeding against the patient's will)
- Seclusion (i.e. restricting the patient from moving freely at the ward, such as locking them in a room)
- Mechanical restraint (i.e. strapping the patient to a belt bed or other device to restrict movement)
- Physical restraint (i.e. staff holding the patient's limbs and thus preventing the patient from moving freely)
- Restricted leave (i.e. preventing the patient from leaving the ward)
- Limiting communications to and from outside the hospital (i.e. limiting letters, access to internet, use of smartphone etc)

- Other:

6. Can coercive measures be used in outpatient care? Please describe.

7. Are there differences in the legislation regarding coercive measures in different age groups?
   - Yes
   - No
   - I don't know

7a. If yes, please describe the differences in as much detail as possible.

SECTION 4: Mechanical restraint

(i.e. strapping the patient to a belt bed or other device to restrict movement)

8. Which legislation allows mechanical restraint in your country?

9. What are the legal requirements to allow mechanical restraint (i.e. what type of behavior must the patient exhibit, duration of restraint, means of restraint use, staff presence etc.)?

10. What is the actual clinical practice concerning mechanical restraint in your country (i.e. what type of behavior must the patient exhibit, duration of restraint, means of restraint use, staff presence etc.)?

SECTION 5: Physical restraint

(i.e. staff holding the patient's limbs and thus preventing the patient from moving freely)

11. Which legislation allows physical restraint in your country?

12. What are the legal requirements to allow physical restraint (i.e. what type of behavior must the patient exhibit, duration of restraint, means of restraint use, staff presence etc.)?

13. What is the actual clinical practice concerning physical restraint in your country (i.e. what type of behavior must the patient exhibit, duration of restraint, means of restraint use, staff presence etc.)?

SECTION 6: Seclusion

(i.e. restricting the patient from moving freely at the ward, such as locking them in a room)

14. Which legislation allows seclusion in your country?

15. What are the legal requirements to allow seclusion (i.e. what type of behavior must the patient exhibit, duration of seclusion, means of seclusion use, staff presence etc.)?

16. What is the actual clinical practice concerning seclusion in your country (i.e. what type of behavior must the patient exhibit, duration of restraint, means of restraint use, staff presence etc.)?

SECTION 7: Duration of coercive measures

17. Are there any general regulations (on state, regional or hospital levels) for the allowed duration of seclusion or restraint (physical, mechanical)?

18. After how long does the measure need to be re-assessed?

19. Is there a maximum allowed duration?

SECTION 8: Involuntary hospitalisation

20. What requirements need to be met for the patient to be involuntarily treated (for instance type of psychiatric illness, need for treatment, risk of harm etc.)?

21. Are there any differences in this legislation between age groups (e.g. children vs. adults)?

- Yes
- No

21a. If yes, please describe the differences in as much detail as possible.

22. Please describe the administrative process for a patient to be involuntarily treated in a psychiatric hospital (for instance who decides that the patient needs to stay in hospital, is the administrative court involved, for how long can the patient stay in hospital involuntarily etc.).

23. What role/rights do parents have if a child or adolescent does not wish to be treated in a psychiatric hospital (e.g. can they legally decide that the minor can be hospitalised)?

24. Can the patient appeal the involuntary hospitalization or other parts of the care?
Yes
No

24a. If yes, please describe what they can appeal and how that works in as much detail as possible.

25. Is outpatient commitment (i.e. compulsory outpatient care/community treatment orders) allowed for the treatment of psychiatric disorders in children and adolescents in your country?

SECTION 9: Additional questions

26. Are there any ethical or practical dilemmas or problems that occur due to the legislation regarding involuntary psychiatric treatment?

- Yes
- No

26a. If yes, please describe.

27. In your field of work do you participate in the process of using coercive measure with children and adolescents (actual "hands-on", making decisions, signing/approving documents, etc.)?

- Yes
- No

27a. If yes, how often do you participate?

- On a daily basis
- On a weekly basis
- On a monthly basis
- A few times per year
- Seldom

28. Do you have any additional comments or suggestions regarding involuntary psychiatric treatment in your country?
